# Wenyang Lishui Decoction Ameliorates Podocyte Injury in Membranous Nephropathy Rat and Cell Models by Regulating p53 and Bcl-2

**DOI:** 10.1155/2020/6813760

**Published:** 2020-05-12

**Authors:** Huan Lu, Yuezhong Luo, Baolin Su, Shuifu Tang, Gangyi Chen, Licai Zhang, Chao Song, Chao Wang, Haitao Tu, Xinbo Wu

**Affiliations:** ^1^Department of Nephrology, The First Affiliated Hospital of Guangzhou University of Chinese Medicine, Guangzhou, Guangdong, China; ^2^Graduate College, Guangzhou University of Chinese Medicine, Guangzhou, Guangdong, China

## Abstract

Wenyang Lishui decoction (WYD) has been frequently used to treat patients with membranous nephropathy (MN) in China. Our previous study *in vitro* showed that WYD aqueous extract could alleviate F-actin reorganization of podocytes induced by serum from idiopathic membranous nephropathy (IMN) patients. This study aims to investigate the effects and molecular mechanisms of WYD on MN. MN rat models were induced by cationic bovine serum albumin. Experimental rats were divided into four groups: normal, model, WYD, and benazepril. The normal group consisted of normal rats receiving distilled water for four weeks, while the model, WYD, and benazepril groups consisted of MN rats receiving distilled water, 16.5 g/kg/day WYD aqueous extract, and 10 mg/kg/day benazepril, respectively. Alanine aminotransferase, kidney function, albumin, and 24 h urine total protein (UTP) were measured. Hematoxylin-eosin and electron microscopy analyses were performed. Mouse podocytes were induced to develop cell models by serum from IMN patients with antibody to the M-type phospholipase A2 receptor and spleen and kidney Yang deficiency syndrome. They were divided into five groups: control, model, 2 mg/ml WYD, 4 mg/ml WYD, and 8 mg/ml WYD. CCK-8 assays, flow cytometry, qRT-PCR, and Western blot analyses were performed. In the animal experiment, side effects of WYD were not found. Also, there was no significant difference in kidney function among the groups. In addition, UTP level was significantly reduced, and kidney histological damage was restored in both WYD and benazepril groups but difference in UTP level between them was not found. In the cell experiment, apoptosis rate was increased in the model group while it was decreased by coincubation with WYD. Besides, mRNA and protein levels of p53 were decreased, and those of Bcl-2 were increased by treatment using WYD. In conclusion, WYD could reduce proteinuria and ameliorate podocyte injury by regulating the expression of p53 and Bcl-2. The study is registered in the Chinese Clinical Trial Registry (ChiCTR-OCH-14005137).

## 1. Introduction

Membranous nephropathy (MN) is one of the most common causes of nephrotic syndrome in adults [1]. MN is caused by immune complex localization in the subepithelial side of the glomerular basement membrane (GBM), and podocytes are the major effector target cell destroyed. To date, the search for antigens in patients with MN has witnessed important breakthroughs. The M-type phospholipase A2 receptor (PLA2R) has been identified as a target antigen in approximately 70% of patients with idiopathic membranous nephropathy (IMN), and the titer of serum antibody to PLA2R (anti-PLA2R) is correlated with disease progression. Despite numerous studies, it is regrettable that there is neither an optimal treatment for MN nor therapeutic approaches available to specifically target podocytes [2].

The podocyte actin cytoskeleton accounts for the shape of podocytes as well as the ability to migrate [3] and is significantly important for podocyte health and disease [4–6]. Previous studies have shown that cytoskeleton reorganization is the common final pathway when podocytes are injured [7]. Therefore, it has been speculated that podocyte actin-targeting interventions must be the ground-breaking therapeutic strategy against kidney diseases [8].

Patients with IMN are clinically characterized by heavy edema and proteinuria. In Chinese medicine theory, the item of IMN is defined as “edema” and the pathology is spleen and kidney Yang deficiency. There is a classic decoction for warming Yang and inducing diuresis to treat edema in Chinese medicine, named Zhen-wu-tang (ZWT). Furthermore, ZWT is widely used by a growing number of physicians to treat many kinds of diseases at home and abroad and has achieved good effects. Experimental studies showed that ZWT could ameliorate proteinuria and has direct effects on inflammatory and oxidative damage in rats [9, 10]. On the basis of ZWT, our senior physicians change the dose of some herbs based on extensive clinical experience and add Huang Qi, which is effective in proteinuria kidney disease, to Wenyang Lishui decoction (WYD) to treat patients with IMN showing spleen and kidney Yang deficiency; they have achieved favorable effects for decades. In addition, our previous study showed that the aqueous extract from WYD could alleviate the reorganization of the mouse podocyte F-actin cytoskeleton injured by serum from patients with IMN with spleen and kidney Yang deficiency [11].

Cytoskeletal alterations are positively correlated with the induction of apoptosis [12]. Podocyte apoptosis is a highly important mechanism in the pathogenesis of many kidney diseases and contributes to the progressive loss of functional renal tissue in chronic kidney disease (CKD) [13, 14]. A previous study showed that aqueous extract from WYD is effective for IMN both *in vitro* and *in vivo*. However, it is not clear whether WYD can influence podocyte apoptosis. Thus, the aim of this study was to explore whether the WYD stabilizes cytoskeletal and cellular functions through reducing podocyte apoptosis and to provide a basis for the development of WYD as a complementary therapy in CKD.

## 2. Materials and Methods

### 2.1. Animals

Male Sprague-Dawley rats (180 ± 20 g) were purchased from the Guangzhou University of Chinese Medicine Research center. The rats were housed under standard conditions of 25 ± 2°C and 55 ± 10% humidity with a 12 h light/12 h dark cycle. The rats were given free access to food and water. After seven days of acclimatization, UTP levels were detected. Rats that were negative for proteinuria were used. Animal experiments were approved by the First Affiliated Hospital of Guangzhou University of Chinese Medicine Ethics Committees (No. ZYYECK【2018】047) and were performed in accordance with national and institutional guidelines.

### 2.2. Cell Cultures

Conditionally immortalized mouse podocyte cell lines were kindly provided by Dr. Danesh (Baylor College of Medicine, USA) and Dr. Wei Shi (Division of Nephrology, Guangdong General Hospital, Guangdong Academy of Medical Sciences, China) and were cultured as described previously. Cells were cultured at 33°C in RPMI-1640 medium (Corning, USA) supplemented with 10% fetal bovine serum (FBS) (Gibco, USA), 1 U/ml recombinant interferon-*γ* (IFN-*γ*) (ProSpec, Israel), and penicillin and streptomycin (Hyclone, USA). After four to five days, at confluence, podocytes were reseeded and incubated at 37°C in culture dishes coated with 12 *μ*g/ml type-I collagen (Corning, USA) in RPMI-1640 medium supplemented with 10% FBS and deprived of IFN-*γ*, for 8 to 14 days to allow differentiation.

### 2.3. Preparation of Serum

Human blood samples were prepared as described previously [15]. A total of 40 in-patients with biopsy-proven IMN were enrolled from January 2014 to September 2015. Of all patients, 31 had spleen and kidney Yang deficiency. Among these 31 patients, 28 were positive for anti-PLA2R, and their fasting venous blood samples at the time of renal biopsy were collected for the subsequent analyses. Blood samples from 30 healthy volunteers were also collected during the same time period. Blood samples were centrifuged at 999 ×g at 4°C for 15 min, and serum was obtained. Then, serum was filtered through a 0.22 *μ*m filter and heated at 56°C for 30 min in a water bath to inactivate complement. All samples were aliquoted and stored at −80°C. This study was approved by the First Affiliated Hospital of Guangzhou University of Chinese Medicine Ethics Committees and written consent was obtained from each participant.

### 2.4. Preparation of the Aqueous Extract of WYD

The aqueous extract of WYD was prepared as described previously [11]. WYD consists of the following six components: 15 g Zhi Fu Zi (*Aconiti Lateralis Radix Praeparata*), 30 g Huang Qi (*Astragalus membranaceus*), 30 g Fu Ling (*Poria*), 45 g Bai Zhu (*Rhizoma Atractylodis Macrocephalae*), 30 g Bai Shao (*Paeoniae Radix Alba*), and 15 g Sheng Jiang (*Zingiberis Rhizoma Recens*). All herbs were purchased from the First Affiliated Hospital of Guangzhou University of Chinese Medicine. All of these herbs were soaked six times with ultrapure water for 30 min. Zhi Fu Zi was decocted for 1 h and then added to the others and boiled at 100°C for 40 min. After pouring out the drug solution, the residue was boiled again. Both the first and the second extractions were collected, filtered, and then centrifuged at 7104 ×g at room temperature for 5 min. The supernatants were collected, concentrated to a final density of 1.65 g raw materials per milliliter, filtered through a 0.45 *μ*m and 0.22 *μ*m filter, respectively, and stored at −20°C. The oxidizability and reducibility of WYD as well as the effect of WYD on cell proliferation were determined by CCK-8 assay according to the manufacturer's method (Dojindo, Japan) as described previously [11].

### 2.5. Animal Experimental Groups and WYD Treatment

The MN rat model was induced by cationic bovine serum albumin (C-BSA) [16] with tail vein injection for four weeks. At the same time, rats in the normal group were given saline. UTP was examined by the turbidimetric method, and renal pathology was detected by electron microscopy to ensure that the model was established successfully. MN rats were randomly divided into model, WYD, and benazepril groups (six rats in each group). During a period of four weeks, the WYD group was orally administered 16.5 g/kg/day WYD aqueous extract according to our previous experiment (data unpublished). The benazepril group was orally administered 10 mg/kg/day benazepril (Novartis, Switzerland, X2606). The normal and model groups were administered an identical volume of distilled water. On day 28, 24 h urine samples were collected using metabolic cages for all rats. Then, rats were anesthetized. Blood samples from the abdominal aorta were collected. After standing at room temperature for 15 min, blood samples were centrifuged at 1360 ×g for 15 min at 4°C to obtain the serum and then stored at −20°C. Animals were then sacrificed, and both kidneys were harvested for histological analysis.

### 2.6. Cell Experimental Groups and WYD Treatment

Podocytes between passages 17 and 22 were used. According to our previous study [11], the working concentration of the serum of patients with IMN inhibited podocyte proliferation by 10%. Before the experiment, podocytes were synchronized into quiescence by starving for 24 h with serum-free RPMI-1640 medium. The experimental groups were designed as follows: (a) for the control group, podocytes were incubated with RPMI-1640 medium containing 10% serum from healthy volunteers; (b) for the model group, podocytes were incubated with RPMI-1640 medium containing 10% serum from patients with IMN; (c) for the 2 mg/ml WYD group, podocytes were incubated with RPMI-1640 medium containing 10% serum from patients with IMN as well as 2 mg/ml WYD aqueous extract; (d) for the 4 mg/ml WYD group, podocytes were incubated with RPMI-1640 medium containing 10% serum from patients with IMN as well as 4 mg/ml WYD aqueous extract; and (e) for the 8 mg/ml WYD group, podocytes were incubated with RPMI-1640 medium containing 10% serum from patients with IMN as well as 8 mg/ml WYD aqueous extract.

### 2.7. Measurement of Liver Function, Kidney Function, and UTP

Serum albumin was measured via the bromocresol green method, and UTP was measured using a turbidimetric method. In addition, the levels of blood urea nitrogen (BUN), serum creatinine (SCr), alanine aminotransferase (ALT), total cholesterol (CHOL), and triglycerides (TG) were also measured with an automatic biochemistry analyzer (Cobas6000, Roche, Switzerland).

### 2.8. Pathology Test of Renal Tissue

For light microscopy analyses, portions of kidney tissues were fixed in 10% neutral formalin phosphate buffer, dehydrated through a graded alcohol series, and embedded in paraffin. The 5 *μ*m thick paraffin sections were stained with hematoxylin-eosin (H&E). For electron microscopy analyses, portions of renal cortex samples were cut into 1 mm cubes on ice, fixed in 2.5% glutaraldehyde immediately, and then fixed in 1% osmium tetroxide (Ted Pella, USA). Both light and electron microscopy (BX41, Olympus, Japan; JEM-1400 PLUS, JEOL, Japan) photographs were blindly evaluated by two experienced pathologists.

### 2.9. Analysis of Apoptosis by Flow Cytometry

Flow cytometry was applied to measure cell apoptosis. Podocytes were starved for 24 h and then plated in 6-well plates at a density of 2 × 10^5^ cells per well. Podocytes were incubated with RPMI-1640 medium containing 10% FBS at 37°C in 5% CO_2_ for 24 h and then treated with serum from healthy volunteers or serum from patients and different concentrations of WYD for 24 h and 48 h, respectively. Annexin V-FITC (BestBio science, China) staining was carried out according to the manufacturer's instructions. Podocytes were harvested with 0.25% trypsin, washed with ice-cold PBS, centrifuged at 916 ×g at 4°C for 4 min, resuspended with binding buffer, and incubated with 5 *μ*l of Annexin V-FITC conjugate for 15 min and 10 *μ*l of propidium iodide solution for 5 min at 4°C in darkness. The cell suspension was analyzed by flow cytometry (Accuri C6, BD biosciences, USA).

### 2.10. Real-Time Quantitative Reverse Transcription-Polymerase Chain Reaction (qRT-PCR) Analysis

Total RNA from cultured podocytes was extracted using an RNA extraction kit (Takara, Japan). Complementary DNA was synthesized from 400 ng of total RNA using PrimeScript™ RT Master Mix (Takara, Japan). RT-PCR was performed on p53, Bcl-2, and GAPDH. The cycling program was set at 40 cycles at 95°C for 5 sec and 56°C for 30 sec after one cycle of predenaturation at 95°C for 30 sec. When the final cycle was over, samples were maintained at 65°C for 5 sec for further extension. The primer (Invitrogen, USA) sequences used for RT-PCR were as follows: p53, forward 5′ GCAACTATGGCTTCCACCTG 3′, reverse 5′ CTCCGTCATGTGCTGTGACT 3′, Bcl-2: forward 5′ ACTGAGTACCTGAACCGGCATCT 3′, reverse 5′ AGCCAGGAGAAATCAAACAGAGG 3′, GAPDH: forward 5′ CCTGGAGAAACCTGCCAAGTATG 3′, reverse 5′ GGTCCTCAGTGTAGCCCAAGATG 3'. Each reaction was amplified in triplicate. The relative levels of the p53 and Bcl-2 mRNAs were the ratio of the absorbance value of p53 and Bcl-2 to that of GAPDH, respectively.

### 2.11. Western Blot Analysis

For isolation of total protein, podocytes were washed with PBS and lysed with Pierce^®^ IP Lysis Buffer (Thermo Scientific, USA) containing 1% Halt Protease Inhibitor Cocktail (Thermo Scientific, USA). The lysate was centrifuged at 13,000 ×g for 10 min at 4°C, and the supernatant was collected. Protein concentrations were measured with a Pierce^TM^ BCA Protein Assay Kit (Thermo Scientific, USA). The equivalent amounts of total protein extracts were diluted with 5x loading buffer and heated in boiling water for 10 min. The proteins were separated by 12% SDS polyacrylamide gels and transferred onto a polyvinylidene difluoride transfer membrane (Millipore, USA) by electroblotting. The membranes were blocked with 5% nonfat dry milk in TBST at room temperature for 1 h and then incubated with primary antibodies (p53 1 : 1000, Bcl-2 1 : 100, Abcam USA; and GAPDH 1 : 1000, Cell signaling, USA) overnight at 4°C. After washing the membranes in TBST four times for 10 min to remove the excess primary antibody, the horseradish-peroxidase-conjugated rabbit anti-mouse IgG or goat anti-rabbit IgG (southern biotech, China) was added and incubated at room temperature for 1 h. The membranes were washed in TBST four times for 10 min and immersed in ECL Plus Western Blot Detection Reagents (Pluslight, Forevergen, China) for 5 min at room temperature. Reproducibility was confirmed in triplicate. The signals were measured with Image-Pro Plus 6.0 software. The level of protein expression was quantified as the ratio of the p53 or Bcl-2 band to the GAPDH band.

### 2.12. Statistical Analyses

Data are reported as the mean ± SD. Data for two groups were analyzed with Student's *t*-test. Data for more than two groups were compared by one-way analysis of variance followed by a Bonferroni *post hoc* test to locate the differences between groups. Differences with a *P* value <0.05 were considered statistically significant.

## 3. Results

### 3.1. Effects of WYD on Metabolic Parameters in MN Rats

**Figure 1:**
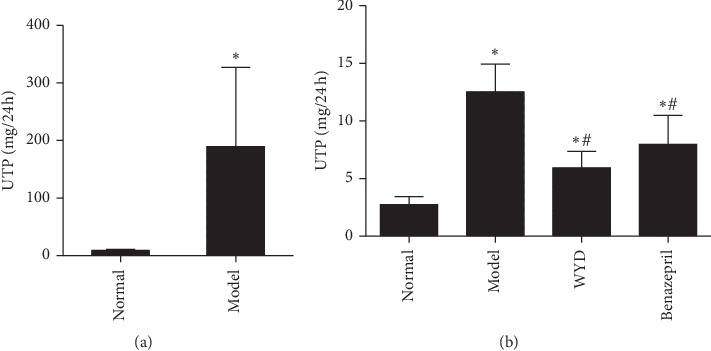
WYD reduced the 24 h proteinuria in MN rats. The MN rat model was induced (a), and WYD reduced the 24 h proteinuria in MN rats at four weeks (b). UTP, 24 h urine total protein; WYD, Wenyang Lishui decoction. The results are expressed as mean ± SD. ^*∗*^*P* < 0.05 vs. the normal group. ^#^*P* < 0.05 vs. the model group.

The metabolic parameters examined after therapy for four weeks are shown in Table 1 and Figure 1. The serum ALB levels were lower and the UTP levels were significantly higher in the model group than in the normal group. Both WYD and benazepril treatment reduced proteinuria. However, no differences in UTP and serum ALB levels were observed between the WYD and benazepril groups. The concentration of TG in the WYD group was the lowest among all groups (*P* < 0.05 vs. model group). WYD treatment increased BUN and SCr levels compared to the normal and benazepril groups but with no statistical significance. Additionally, the level of ALT did not change after treatment. These data indicated that both WYD and benazepril had no toxic side effects and were safe for the liver and kidney.

### 3.2. Effects of WYD on Renal Tissue Damage in MN Rats

**Figure 2:**
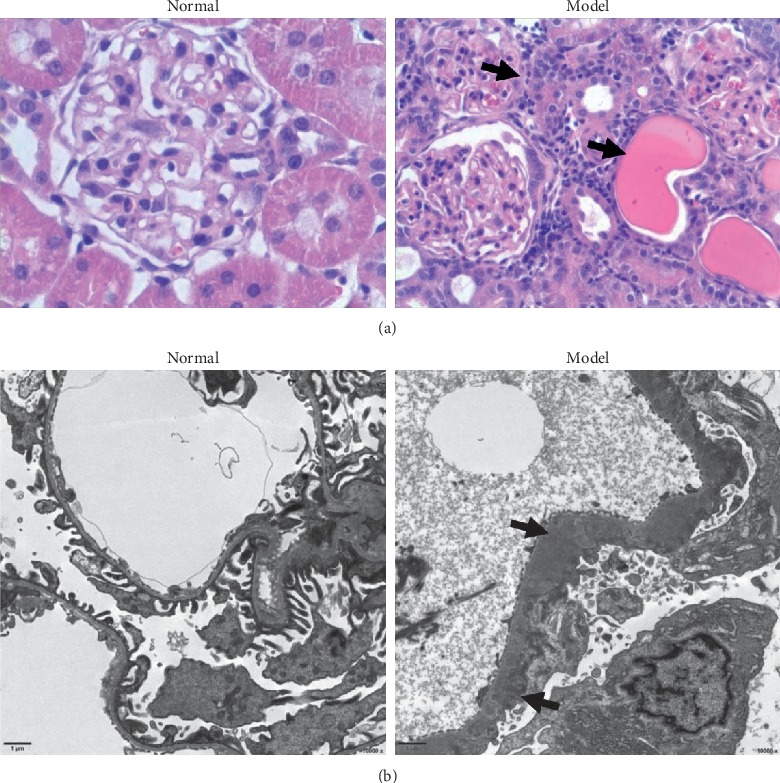
Diffused thickness of GBM and marked podocyte foot process effacement in MN rats. (a) H&E staining of renal tissue shows the appearance of glomeruli and tubules from the normal and model groups (400x). The arrows represent the inflammatory infiltration in the interstitium and protein casts in the tubules. (b) Electron microscopic images of glomeruli show diffused GBM thickening, effacement of the podocyte foot process, and electron dense deposits in the subepithelium (black arrows) in the model group. Scale bar, 1 *μ*m. Original magnification: 10,000x. GBM, glomerular basement membrane. H&E, hematoxylin-eosin.

**Figure 3:**
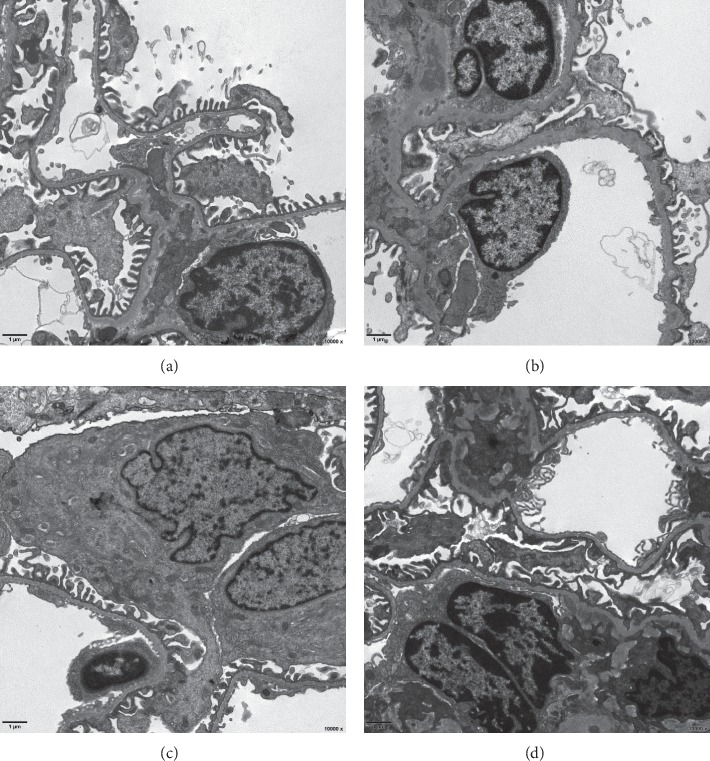
Effects of WYD on renal tissue damage in MN rats. Compared to the model group, the diffused thickness of the GBM and podocyte foot process effacement were obviously ameliorated in the WYD group and benazepril group. Scale bar, 1 *μ*m. Original magnification: 10,000x. WYD, Wenyang Lishui decoction. GBM, glomerular basement membrane. (a) Normal. (b) Model. (c) WYD. (d) Benazepril.

The results of renal histopathology detection are shown in Figures 2 and 3. After C-BSA injection for four weeks, electron microscopy detected diffused thickness of the GBM and marked podocyte foot process effacement in the model group. In addition, protein casts, tubular epithelial cell edema, and inflammatory infiltration were also observed in the model group rats. After treatment with WYD for four weeks, the diffused thickness of the GBM and podocyte foot process effacement were obviously ameliorated. Renal tissue damage was also relieved in the benazepril group.

### 3.3. WYD Decreased the Apoptosis of Podocytes Incubated with the Serum of Patients with IMN

**Figure 4:**
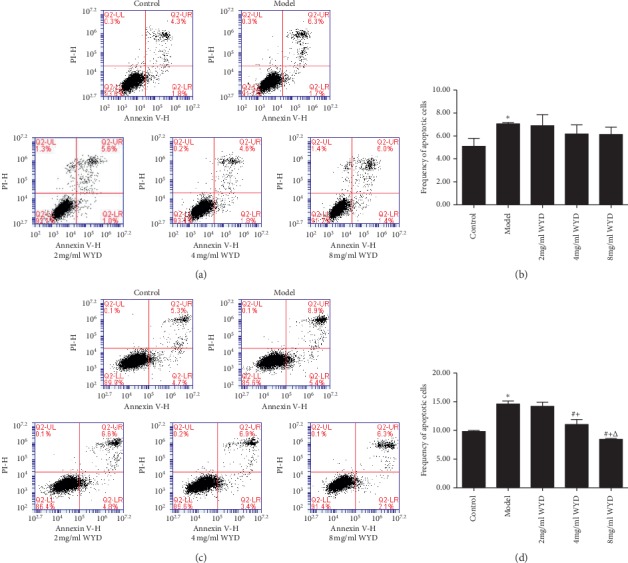
WYD protects podocytes from apoptosis in the MN cell model. Podocytes were incubated with RPMI-1640 medium containing serum from volunteers or serum from patients and different concentrations of WYD for 24 h (a and b) and 48 h (c and d). (a and c) Flow cytometry dot plots of podocytes stained with Annexin V- FITC and propidium iodide. (b and d) Bar graphs show the results of the podocyte apoptosis rate (%) in each group. All values are expressed as mean ± SD. WYD, Wenyang Lishui decoction. ^*∗*^*P* < 0.05 vs. the control group. ^#^*P* < 0.05 vs. the model group. ^+^*P* < 0.05 vs. the 2 mg/ml WYD group. ^Δ^*P* < 0.05 vs. the 4 mg/ml WYD group.

To determine the effect of WYD on podocyte apoptosis, Annexin V-FITC and propidium iodide were used. Podocytes were cultured in RPMI-1640 medium containing the serum of volunteers or patients and different concentrations of WYD for 24 h and 48 h, respectively. As shown in Figure 4, the number of apoptotic cells was clearly higher in the model group than in the control group and decoction groups. Additionally, the difference in the cell apoptotic rate in the control group and model group was statistically significant for both 24 h and 48 h (*P* < 0.05). For the decoction groups, we observed a concentration-dependent relationship with regard to the WYD and apoptotic cells (Table 2). The percentage of apoptotic cells in the 8 mg/ml WYD group was the lowest and showed significant differences among different concentrations of WYD for 48 h (*P* < 0.05).

### 3.4. Effects of WYD on Gene Expression Related to Apoptosis

**Figure 5:**
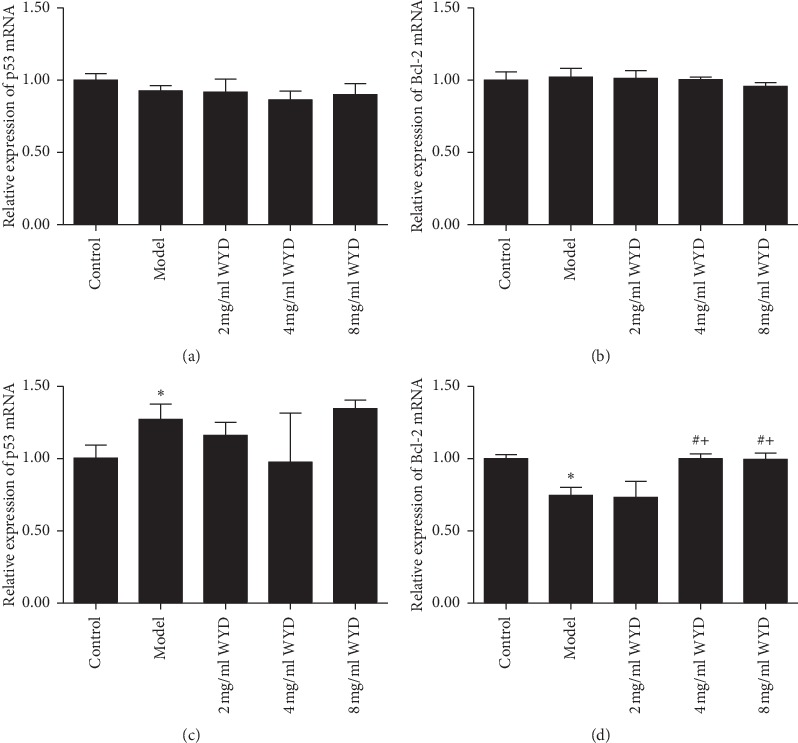
Effects of WYD on p53 and Bcl-2 mRNA expression in the MN cell model. Bar graphs show the expression of p53 and Bcl-2 mRNA in each group. Podocytes were incubated with RPMI-1640 medium containing serum from volunteers or serum from patients and different concentrations of WYD for 24 h (a and b) and 48 h (c and d). WYD, Wenyang Lishui decoction. ^*∗*^*P* < 0.05 vs. the control group. ^#^*P* < 0.05 vs. the model group. ^+^*P* < 0.05 vs. the 2 mg/ml WYD group.

The mRNA expression of p53 and Bcl-2 was also detected by qRT-PCR. As shown in Figure 5, the relative mRNA expression of p53 was obviously higher in the model group than in the control group and showed a significant difference for 48 h (*P* < 0.05). Compared to the model group, the mRNA expression of p53 in the decoction groups was downregulated. However, there was no statistically significant difference between the model group and the decoction groups (*P* > 0.05). In addition, the changes in the decoction groups showed no significant differences compared to each other (*P* > 0.05). The mRNA expression of Bcl-2 was reduced in the model group compared to that in the control group and showed a significant difference only for 48 h (*P* < 0.05). Similar to the apoptosis results, a concentration-dependent relationship with regard to the WYD and the mRNA expression of Bcl-2 was observed for 48 h. Compared to the model group, the mRNA expression of Bcl-2 in the 4 mg/ml and 8 mg/ml WYD groups was upregulated and showed a significant difference for 48 h (*P* < 0.05).

### 3.5. Effects of WYD on Protein Expression Related to Apoptosis

**Figure 6:**
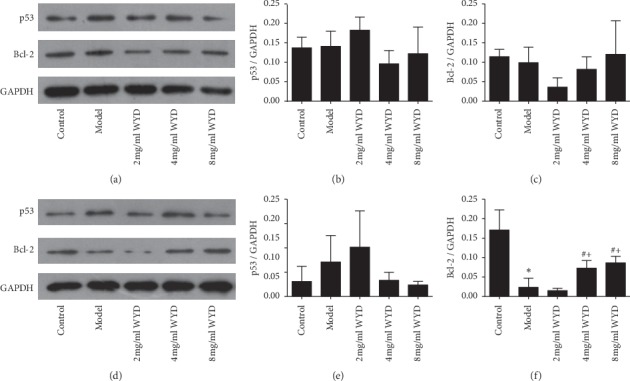
Effects of WYD on p53 and Bcl-2 protein expression in the MN cell model. Podocytes were incubated with RPMI-1640 medium containing serum from volunteers or serum from patients and different concentrations of WYD for 24 h (a, b, and c) and 48 h (d, e, and f). (a and d) The protein levels of p53 and Bcl-2 were analyzed using Western blot. For densitometry, GAPDH was used as an internal control. A reduction in p53 expression and an increase in Bcl-2 expression were detected in the 4 mg/ml and 8 mg/ml WYD groups compared with the model group both for 24 h and 48 h incubation. (b, c, e, and f) Bar graphs show the expression of p53 and Bcl-2. WYD, Wenyang Lishui decoction. ^*∗*^*P* < 0.05 vs. the control group. ^#^*P* < 0.05 vs. the model group. ^+^*P* < 0.05 vs. the 2 mg/ml WYD group.

To explore the mechanisms responsible for the inhibitory effects of WYD on the apoptosis of podocytes incubated with serum from patients with IMN, the protein expression of p53 and Bcl-2 was investigated. As shown in Figure 6, the relative expression of p53 was clearly higher in the model group than in the control group both for 24 h and 48 h. However, there was no statistically significant difference between them (*P* > 0.05). Compared to the model group, the relative expression of p53 in the 4 mg/ml and 8 mg/ml WYD groups was downregulated, whereas, in the 2 mg/ml WYD group, it was upregulated. However, there was no statistically significant difference between the model group and decoction groups. Furthermore, the changes in the decoction groups showed no significant differences compared to each other (*P* > 0.05). The relative expression of Bcl-2 was reduced in the model group. Moreover, there was a statistically significant difference between the model group and the control group for 48 h (*P* < 0.05). Similar to the apoptosis results, a concentration-dependent relationship with regard to WYD and the relative expression of Bcl-2 was observed for 24 h and 48 h. Compared to the model group, the relative expression of p53 in the 4 mg/ml and 8 mg/ml WYD groups was upregulated and showed a significant difference for 48 h (*P* < 0.05).

## 4. Discussion

To date, the exact mechanism of IMN pathology is unclear, and there is no effective cure for the disease. Although podocyte structure is restored and proteinuria resolves in response to glucocorticoids combined with other immunosuppressive agents, sometimes there is still a proportion of patients with IMN who are unresponsive to immunosuppressive therapy or who experience frequent recurrence and rapidly develop end-stage renal disease. In addition, the side effects of immunosuppressive treatments often have a bad effect on patients' health as well as disease progression. Approximately one-third of these patients will develop spontaneous remission. How and when to treat patients with costly and potentially toxic drugs remains a challenge to nephrologists [17]. Chinese herbs are frequently used to treat acute and chronic glomerular disease and exert great influence on patients in China. There is an increasing body of literature suggesting that warming Yang and inducing diuresis has a good effect on IMN. However, the exact mechanism of this therapy is unclear. In this study, C-BSA-induced MN rat models were developed *in vivo*. With oral administration of WYD for four weeks, UTP was reduced and kidney histological damage was restored. A conditionally immortalized mouse podocyte cell line was used to establish an IMN model *in vitro*. After incubation with 2 mg/ml, 4 mg/ml, and 8 mg/ml WYD, the F-actin cytoskeleton structure of podocytes was stabilized [11], and cell apoptosis was attenuated.

To investigate the pathology of many kinds of kidney disease, serum from patients was incubated with kidney sections or renal intrinsic cells *in vitro*. Bitzan et al. [18] collected blood plasma samples from patients with recurrent or primary FSGS and incubated the samples with normal human podocytes *in vitro*, leading to disturbance of the cell cytoskeleton and *β*3 integrin-mediated podocyte attachment. A study by Harris et al. [19] used nephrotic plasma from patients with FSGS, incubated the plasma with human conditionally immortalized podocytes, and demonstrated a specific biomarker for disease activity. It is necessary to further test potential patient blood and cell-based assays of disease mechanisms. As anti-PLA2R is central to the disease, serum collected from patients at the onset of disease and with positive serum anti-PLA2R is required. The model was developed by incubating podocytes with medium containing anti-PLA2R-positive sera from patients with IMN with spleen and kidney Yang deficiency syndrome in this study. The concentration of IMN serum used was determined based on our previous data [11] that 10% of patient serum was able to obviously decrease the ability of podocytes to proliferate. After coincubation for 24 h, the injured structural integrity of the cytoskeleton, decreased cell proliferation ability, and increased apoptosis rate in the model group indicated that the model of podocyte injury induced by serum from patients with IMN was successfully established in our study.

The cytoskeleton is central for distinct functions. It is known that the actin cytoskeleton is a major regulator of cellular stiffness and is important for a normal podocyte mechanical phenotype and cellular function. The actin cytoskeleton could be affected in glomerular disease [20, 21]. A growing number of papers have shown that the cytoskeleton is injured by oxidative stress and can be restored by Chinese herbs [22, 23]. Moreover, Chinese herbs also have a positive effect on the podocyte cytoskeleton. A study by Zheng et al. [24] using astragaloside IV for therapy demonstrated that astragaloside IV could restore the actin cytoskeleton damaged by the complement membranous attack complex and attenuate podocyte injury in MN. Sai et al. [25] found that cotreatment with astragalosides inhibited the rearrangement and destruction of the cytoskeleton in the mouse podocyte clone 5 (MPC5) podocytes with adriamycin-induced damage. Consistent with those prior studies, we found that WYD could stabilize the structure of the F-actin cytoskeleton in immortalized mouse podocyte cell lines destroyed by sera from patients with IMN [11]. It is well known that apoptosis induction is closely related to changes in the actin cytoskeleton [26, 27]. Whether WYD alleviates podocyte injury by adjusting cell apoptosis was further evaluated.

Mounting evidence has accumulated to suggest that Chinese herbal medicines had inhibitory effects on cell apoptosis both *in vitro* and *in vivo* [28–31]. Gui et al. [28] demonstrated that astragaloside IV acts directly on podocytes and prevents glucose-induced podocyte apoptosis by increasing Bcl-2 expression and decreasing Bax expression. WYD consists of Huang Qi and five other components. In the present study, we found that the serum of patients with IMN could increase podocyte apoptosis as detected by flow cytometric analysis. After incubation with WYD, the apoptosis rate was decreased. In addition, we found that the mRNA and protein expression levels of Bcl-2 were decreased in podocytes incubated with serum from patients with IMN, and this effect was depressed after cotreatment with WYD for 48 h. Similarly, the mRNA and protein expression levels of p53 were also affected by WYD between the model group and herb group. Interestingly, WYD prevented serum-induced podocyte apoptosis by increasing Bcl-2 expression and decreasing p53 expression. These results were consistent with the flow cytometric analysis results.

There are some results that were different from those of other studies. First, the levels of gene expression and the relative protein were not consistent. On the one hand, the total level of target protein rather than phosphorylation or ubiquitination protein was examined. However, gene expression is affected by many factors. Evidence suggests that there is a link between metabolism and epigenetic modifying enzymes that makes gene expression more complicated [32]. On the other hand, Chinese formulas consist of many kinds of herbs and have numerous targets. Although we have evaluated the effect of WYD on apoptosis, it is likely that WYD may also operate through other mechanisms. Second, a dose-dependent effect was not observed among the WYD treatment groups *in vitro* for every target index. It is well known that many acupoints have bidirectional effects in acupuncture. Moreover, many herbs also have different effects when different doses are given. For example, *Bupleurum chinense* could relieve superficies syndrome in a small dose while reconciling *Shao Yang* in a large dose.

## 5. Conclusions

Overall, we explored the effect of WYD on C-BSA-induced MN rats and whether WYD could ameliorate damage of immortalized mouse podocytes that were coincubated with serum from patients with IMN who were positive for anti-PLA2R and with spleen and kidney Yang deficiency. Oral administration of WYD or benazepril for four weeks to MN rats could reduce UTP and restore kidney histological damage. Coincubation with WYD decreased podocyte apoptosis. As a possible mechanism, WYD may modulate apoptosis by increasing the expression of Bcl-2 and decreasing p53. Therefore, these results suggest that WYD could be considered an effective complementary treatment for MN.

## Figures and Tables

**Table 1:** Effects of WYD on metabolic parameters in MN rats.

Group	BUN (mmol/L)	SCr (*μ*mol/L)	ALB (mmol/L)	TG (mmol/L)	CHOL (mmol/L)	ALT (U/L)
Normal	4.69 ± 0.16	40.33 ± 2.08	37.10 ± 0.52	0.67 ± 0.16	1.29 ± 0.4	27.67 ± 4.73
Model	4.03 ± 0.95^∗^	41.00 ± 5.94	35.08 ± 1.3	0.70 ± 0.1^∗^	2.07 ± 0.42	42.75 ± 17.65
WYD	6.57 ± 0.69^#^	45.25 ± 3.1	36.23 ± 1.96	0.44 ± 0.17^#^	1.73 ± 0.29	30.25 ± 2.99
Benazepril	4.70 ± 1.26	38.75 ± 5.19	36.00 ± 2.29	0.48 ± 0.13	2.32 ± 0.63^∗^	42.75 ± 12.58

BUN, blood urea nitrogen; SCr, serum creatinine; ALB, albumin; UTP, 24 h urine total protein; TG, triglycerides; CHOL, total cholesterol; ALT, alanine aminotransferase; WYD, Wenyang Lishui decoction. ^*∗*^*P* < 0.05 vs. the normal group. ^#^*P* < 0.05 vs. the model group.

**Table 2:** Comparison in cell apoptosis rate (mean ± SD).

Groups	Incubation times	
	24 h	48 h
Control	5.1000 ± 0.6928	9.8333 ± 0.1528
Model	7.0667 ± 0.1155^∗^	14.600 ± 0.5196^∗^
2 mg/ml WYD	6.9000 ± 0.9644	14.2000 ± 0.7211
4 mg/ml WYD	6.1667 ± 0.8083	11.0333 ± 0.8737^# +^
8 mg/ml WYD	6.1333 ± 0.6351	8.4333 ± 0.1528^# +△^

WYD, Wenyang Lishui decoction. ^*∗*^*P* < 0.05 vs. the control group. ^#^*P* < 0.05 vs. the model group. ^+^*P* < 0.05 vs. the 2 mg/ml WYD group. ^Δ^*P* < 0.05 vs. the 4 mg/ml WYD group.

## Data Availability

The datasets used and analyzed during the current study are available from the corresponding author upon reasonable request.
